# The Scottish BioHermes Data Challenge: democratising dementia data access

**DOI:** 10.1002/alz70856_101431

**Published:** 2025-12-25

**Authors:** Kalliopi Mavromati

**Affiliations:** ^1^ University of Glasgow, Glasgow, Scotland, United Kingdom

## Abstract

**Background:**

Direct access to individual participant data (IPD) from completed research studies is an aspiration, but frequently ‘open’ data are not equally open to all in the research community. Through a partnership of Scottish Brain Health Alliance for Research Challenges, Global Alzheimer's Platform Foundation (GAP), and the Alzheimer's Disease (AD) Data Initiative, we attempted to truly democratise data access, creating The Scottish Data Challenge data sharing initiative.

**Method:**

We used the GAP BioHermes dataset, containing clinical, demographic and test data, including a variety of novel dementia biomarkers, from over 1000 participants. The cohort is uniquely drawn from populations typically underrepresented in dementia research. Hosted on the AD Data Initiative's AD Workbench, we made the BioHermes IPD available to teams wishing to explore new hypotheses. We ensured ethics and data governance were in place to minimise administrative burden, and offered end‐to‐end support from developing the research question, to running analyses and dissemination. The only request of applicants was to prioritise collaboration, support early career researchers and consider equality, diversity, and inclusion in the composition of their teams.

**Result:**

Following expressions of interest, more than 40 international teams, comprising over 100 individual researchers from multidisciplinary backgrounds have accessed the resource. Active projects include describing the effect of frailty on dementia risk, incorporating biomarkers into cognitive reserve models, and assessing the moderating effect of cardiovascular risk factors. Teams will complete their analyses and share results at a research showcase event in March 2025. We will highlight results from some of the prize winning projects as part of this symposium.

**Conclusion:**

By allowing researchers to focus on what they do best – research, our easy access, open data resource succeeded in facilitating innovation through collaboration. The quality and volume of results produced in the few months since the launch are testament to the potential of open data. We hope the Challenge will act as a model for future data sharing initiatives.

